# Inequalities in child mortality in ten major African cities

**DOI:** 10.1186/1741-7015-12-95

**Published:** 2014-06-06

**Authors:** Wilm Quentin, Olayinka Abosede, Joseph Aka, Patricia Akweongo, Kouassi Dinard, Alex Ezeh, Ramadan Hamed, Patrick Kalambayi Kayembe, Getnet Mitike, Gemini Mtei, Marguerite Te Bonle, Leonie Sundmacher

**Affiliations:** 1 Department of Health Care Management and Berlin Centre for Health Economics Research (BerlinHECOR), Technische Universität (TU) Berlin, Straße des 17. Juni 135, Berlin 10623, Germany; 2 Primary Health Care & Nutrition Unit, College of Medicine of the University of Lagos, PMB, Lagos 12003, Nigeria; 3 Institut National de Santé Publique, Abidjan BPV 47, Côte d’Ivoire; 4 School of Public Health, University of Ghana, P.O. Box LG 13, Legon, Ghana; 5 African Population and Health Research Centre, P.O. Box 10787–00100, Nairobi, Kenya; 6 Social Research Centre, American University in Cairo, El Tajomee El Khames, New Cairo, Egypt; 7 Ecole de Santé Publique, Université de Kinshasa, BP 11850, Kinshasa 1, Democratic Republic of the Congo; 8 School of Public Health, Addis Ababa University, Addis Ababa, Ethiopia; 9 Ifakara Health Institute, Plot 463, Kiko Avenue, Mikocheni, Off Mwai Kibaki Road, P.O. Box 78373, Dar es Salaam, Tanzania; 10 Department of Health Services Management, Munich School of Management, Ludwig-Maximilians-Universität, Schackstraße 4, Munich 80539, Germany

**Keywords:** Socioeconomic factors, Urban health, Child mortality, Africa, Social justice

## Abstract

**Background:**

The existence of socio-economic inequalities in child mortality is well documented. African cities grow faster than cities in most other regions of the world; and inequalities in African cities are thought to be particularly large. Revealing health-related inequalities is essential in order for governments to be able to act against them. This study aimed to systematically compare inequalities in child mortality across 10 major African cities (Cairo, Lagos, Kinshasa, Luanda, Abidjan, Dar es Salaam, Nairobi, Dakar, Addis Ababa, Accra), and to investigate trends in such inequalities over time.

**Methods:**

Data from two rounds of demographic and health surveys (DHS) were used for this study (if available): one from around the year 2000 and one from between 2007 and 2011. Child mortality rates within cities were calculated by population wealth quintiles. Inequality in child mortality was assessed by computing two measures of relative inequality (the rate ratio and the concentration index) and two measures of absolute inequality (the difference and the Erreyger’s index).

**Results:**

Mean child mortality rates ranged from about 39 deaths per 1,000 live births in Cairo (2008) to about 107 deaths per 1,000 live births in Dar es Salaam (2010). Significant inequalities were found in Kinshasa, Luanda, Abidjan, and Addis Ababa in the most recent survey. The difference between the poorest quintile and the richest quintile was as much as 108 deaths per 1,000 live births (95% confidence interval 55 to 166) in Abidjan in 2011–2012. When comparing inequalities across cities or over time, confidence intervals of all measures almost always overlap. Nevertheless, inequalities appear to have increased in Abidjan, while they appear to have decreased in Cairo, Lagos, Dar es Salaam, Nairobi and Dakar.

**Conclusions:**

Considerable inequalities exist in almost all cities but the level of inequalities and their development over time appear to differ across cities. This implies that inequalities are amenable to policy interventions and that it is worth investigating why inequalities are higher in one city than in another. However, larger samples are needed in order to improve the certainty of our results. Currently available data samples from DHS are too small to reliably quantify the level of inequalities within cities.

## Background

The existence of health-related inequalities across different areas as well as across different socio-economic groups is well documented [1-5]. Numerous studies have demonstrated that urban areas have lower infant and child mortality rates than rural areas [3,4,6-9]; and there is even more extensive literature showing that mortality is much lower among higher socio-economic groups than among the poor [2,10-14].

African cities are growing faster than cities in most other regions of the world [15], and by 2020 more people are estimated to be living in African cities than in European cities. In 2010, almost 40% of Africans lived in urban areas, with about 33% of urban Africans living in cities of more than 1 million inhabitants [15]. Living conditions in Sub-Saharan African cities remain particularly poor, and more than 60% of city dwellers live in slums [16], meaning that they lack either durable housing, sufficient space, access to safe water or basic sanitation. In addition, economic inequality as measured by the Gini-coefficient is higher in African cities than in most other cities of the world [17]. In Northern African cities, living conditions are better with about 13% of the urban population living in slums [16].

In 2010, child (under-five years old) mortality in Africa was estimated to be at 111 per 1,000 live births [18]. On average 132 children died per 1,000 live births in rural areas compared to 102 in urban areas (based on data from 28 African countries with recent (2005 to 2011) data from demographic and health surveys (DHS)) [19]. However, while child mortality is on average lower in urban areas, a previous study and a World Health Organization (WHO) report have highlighted that child mortality of the poor is often higher in urban than in rural areas [20,21]; this is explained by the fact that inequalities between rich and poor tend to be larger in cities than in rural areas.

Inequalities in child mortality are generally considered to constitute inequities because they are perceived to be unfair, socially produced and potentially modifiable [22]. A 2010 joint WHO and United Nations Human Settlements Programme (UN-HABITAT) report [21] urged countries to disaggregate data within cities in order to unmask health-related inequities. However, available studies of urban inequalities in child mortality usually look at all urban residents within a country [7,9,20,23]. Only very few studies are available that investigate inequalities within specific African cities [24-26], and systematic information comparing the magnitude of inequalities in child mortality across different cities and the development of inequalities over time is unavailable. Yet, unless inequalities are investigated and revealed, it is impossible for governments to act against them [21].

This study aimed to systematically compare inequalities in child mortality across 10 major African cities and to investigate the development of inequalities over time (the last decade or so, depending on the availability of data). More specifically, the objectives were: (1) to calculate child mortality rates by wealth quintiles within cities; (2) to quantify the degree of inequality within cities using different established measures of inequality [27,28]; (3) to determine whether child mortality is more unequally distributed in some cities than in others; and (4) to assess whether certain cities have succeeded in reducing inequalities over time.

## Methods

### Data availability and city sample

Data for this study were drawn from DHS and one malaria indicator survey (MIS) (see Table 1). These surveys are generally carried out by national statistical offices with support provided by Measure DHS, a project that helps developing countries collect data on health and population trends [29]. Surveys and datasets are highly standardized and inconsistencies in responses are reduced during data processing [30]. Among many other things, each included survey contains information on the complete birth histories of interviewed women (15- to 49- years old), that is, information on dates of all births and deaths of their children, and an indicator of household living standards in the form of a wealth index [31].

**Table 1:** Population size of cities included in the sample and characteristics of surveys available for these cities

	**Population**			**Earlier surveys (n in city)**			**More recent surveys (n in city)**		
**City, country**	**City (thousands)**	**Country (thousands)**	**% in city**	**Name, year**	**n births**^**a**^	**n deaths <5**^**a**^	**Name, year**	**n births**^**a**^	**n deaths <5**^**a**^
Cairo, Egypt	11,031	78,076	14%	DHS 2000	1281	44	DHS 2008	1,109	43
Lagos, Nigeria	10,788	159,708	7%	DHS 2003	311	25	DHS 2008^b^	1,158	92
Kinshasa, DRC	8,415	62,191	14%	-	-	-	DHS 2007	1,708	146
Luanda, Angola	4,790	19,549	25%	-^c^	-	-	MIS 2011	2,873	181
Abidjan, Côte d’Ivoire	4,151	18,977	22%	DHS 1998-99	1059	112	DHS 2011-12	1,204	100
Dar es Salaam, Tanzania	3,415	44,973	8%	DHS 1999	295	32	DHS 2010^d^	323	30
Nairobi, Kenya	3,237	40,909	8%	DHS 1998	361	21	DHS 2008-09	730	40
Dakar, Senegal	2,926	12,951	23%	DHS 1997	1145	86	DHS 2010-11	1,162	58
Addis Ababa, Ethiopia	2,919	87,095	3%	DHS 2000	1042	120	DHS 2011	832	41
Accra, Ghana	2,469	24,263	10%	DHS 1998	432	18	DHS 2008^e^	365	16

^a^In years 0 to 9 prior to survey; ^b^DHS 2008 was selected instead of MIS 2010 because the 2008 sample was much larger; after verification with GPS coordinates, a non-city cluster of households was excluded; ^c^data in an earlier MIS survey (2006 to 2007) overlaps completely with the data in MIS 2010 as only data on children born in the last six years are included; ^d^DHS 2010 was selected instead of DHS 2011 to 2012 as the 2010 sample was much larger; ^e^after verification with GPS data, several clusters of households located in Tema were excluded. DHS, demographic and health surveys; GPS, global positioning system; MIS, malaria indicator survey.

The 10 largest cities in Africa according to data from the UN World Urbanization Prospects (2011 Revision) [15] were included in the city sample if they fulfilled the following two criteria: (1) they were the largest city in their country (that is, only one city per country was included); and (2) there was at least one DHS conducted after 2005. DHS data were downloaded from the Measure DHS website [32]. In addition, for each city, data from one survey that was closest to the year 2000 and that contained a wealth index variable was also included in the data sample in order to enable trend comparisons.

Table 1 summarizes information about the selected sample of cities and the available data. Cities included in the sample had between 2.5 million (Accra) and 11.0 million (Cairo) inhabitants in 2010, and almost always accounted for a sizeable part of the total population in their respective countries, that is, between 3% (Addis Ababa) and 25% (Luanda). For eight cities, data are available from a survey conducted around the year 2000 (between 1997 and 2003). Four of these surveys contain full birth histories on more than 1,000 births having occurred in the 10 years prior to the interview, while the other four surveys have a much smaller sample size (less than 500 births). The number of deaths of children under-five years of age that occurred in the samples ranges from 18 in Accra to 112 in Abidjan. The more recent surveys were conducted between 2007 (Kinshasa) and 2011–2012 (Abidjan). Most of these later surveys include full birth histories of more than 1,000 children and only two surveys contain fewer than 500 children. The number of included under-five deaths ranges from 16 in Accra to 181 in Luanda.

### Calculation of child mortality by wealth quintile

Household living standards are measured in DHS surveys using information on, among others, ownership of selected assets (for example, bicycle, phone), availability of basic services (for example, water supply, electricity) and household flooring material [31]. This information is aggregated into a household wealth index score, which places households on a continuous scale of relative wealth, using principal component analysis. For this study, the wealth index score available in DHS data sets was used to create city-specific wealth quintiles of births, by ordering all included births (those having occurred in the city in the previous 10 years) according to their wealth index score and dividing the distribution at the cut-off points of each 20 percent section. Sample weights were not used in the analyses because households within cities had the same probability of being selected into the DHS samples. (Please see Additional file 1: Box S1 in the supplementary online material for a justification.)

Child (under-five years old) mortality rates were calculated separately for each wealth quintile using a synthetic cohort life table approach as is standard practice for DHS analyses [29]. The synthetic cohort life table method combines mortality probabilities for small age segments, for which real cohort mortality can be calculated, into total child mortality using a life table approach. Following DHS methodology [29], mortality probabilities were calculated for eight age segments (0, 1 to 2, 3 to 5, 6 to 11, 12 to 23, 24 to 35, 36 to 47, 48 to 59 months), and the total child mortality rate was calculated as the cumulative probability of having died at the end of the last age segment. Calculations were performed in SPSS Version 15. Ninety-five percent confidence intervals around child mortality rates were calculated from standard errors of the cumulative probability of dying.

### Assessment of inequalities

Inequality across wealth quintiles was visualized by drawing concentration curves [33-35]. The child mortality concentration curve plots the cumulative proportion of deaths (on the y-axis) against the cumulative percentage of children at risk ranked by wealth quintile (on the x-axis), beginning with the poorest and ending with the richest [34]. If all children, irrespective of the wealth status of their household, had exactly the same mortality rates, the concentration curve would be a 45-degree line, running from the bottom left-hand corner to the top right-hand corner. If more children die in the poorer quintiles than in the richer quintiles, the concentration curve lies above this line of equality.

In order to assess the level and development of inequalities across cities, two simple and two more complex measures of inequality were calculated [27]: the difference, the ratio, the concentration index and the Erreygers index [28,36]. The difference in child mortality rates between the richest and the poorest quintiles was calculated because it is the most straightforward measure of absolute inequality, and the ratio of the child mortality rate in the poorest divided by the rate in the richest quintile because it is the simplest relative measure of inequality [27]. Confidence intervals for the rate difference and the rate ratio were calculated according to methods described by Moser *et al*. [14].

The concentration index was calculated to quantify the magnitude of relative inequality across all wealth quintiles [27,35]. The concentration index is defined as twice the area between the concentration curve and the line of equality [33,34,37] and has become the standard tool in health economics for evaluating socioeconomic inequalities [36]. In addition, because the value of the concentration index is affected by the average level of child mortality in a city [38], a normalized version of the concentration index, that is, the Erreygers index was calculated [39]. While the concentration index (being a relative measure of inequality) would indicate that the same absolute difference in child mortality between the rich and poor is more severe when child mortality is low, the Erreygers index would show no change in inequality if child mortality of all wealth quintiles was increased (or reduced) by the same absolute amount [36]. Furthemore, the Erreygers index has the advantage that the level of measured inequality would be the same, independent of whether child mortality or child survival was measured [28,39]. Confidence intervals around concentration indices and Erreygers indices were calculated according to methods described by Kakwani *et al*. [35,37].

## Results

### Child mortality by wealth quintiles across cities

**Figure 1:**
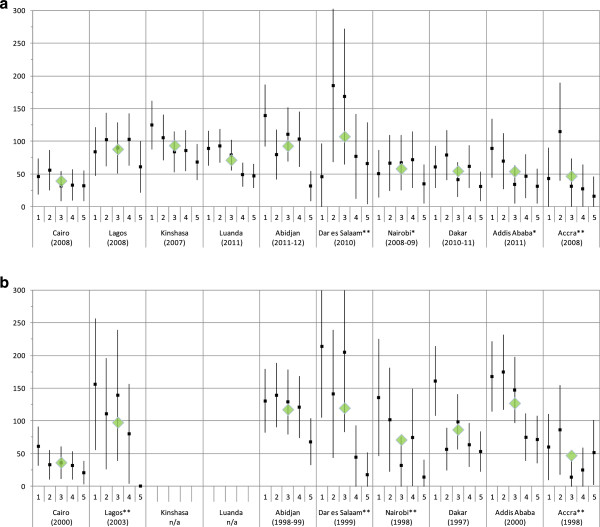
**Under-five mortality rates by wealth quintiles for 10 African cities, development over time. a**: Mean under-five mortality rates (diamonds) and rates by wealth quintiles (with 95% confidence intervals) in ten African cities, more recent surveys. Notes: Quintile 1 = poorest; quintile 5 = richest; * sample size <1,000 children; ** sample size <500 children. **b**: Mean under-five mortality rates (diamonds) and rates by wealth quintiles (with 95% confidence intervals) in eight African cities, earlier surveys. Notes: Quintile 1 = poorest; quintile 5 = richest; ** sample size <500 children.

Figure 1a shows mean child (under-five years old) mortality rates and rates by wealth quintiles across the ten cities included in the more recent round of surveys. Mean child mortality rates range from about 39 deaths per 1,000 live births in Cairo (2008) to about 107 deaths per 1,000 live births in Dar es Salaam (2010). Because of relatively small sample sizes, point estimates of child mortality rates by wealth quintiles are associated with considerable uncertainty as indicated by large 95% confidence intervals. However, except for Dar es Salaam, where the sample size is particularly small, the richest quintiles always have the lowest child mortality rates, while poorer quintiles have considerably higher rates. In addition, it is clear that the size of the difference between rich and poor and the pattern of the distribution of child mortality rates across quintiles vary across cities, even when considering the large degree of uncertainty.Figure 1b shows child mortality rates calculated from the earlier surveys. Mean child mortality rates in most cities were even higher at the time of the earlier surveys. The degree of uncertainty around point estimates in several cities is even larger than in the later surveys. Nevertheless, the difference between rich and poor appears to have been even greater in these surveys.

### Development of inequalities within cities over time

**Figure 2:**
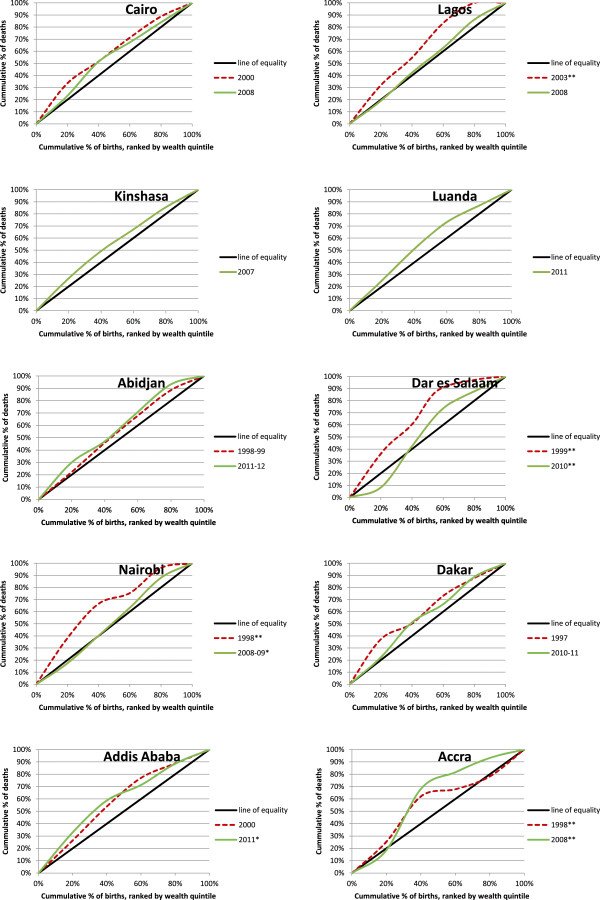
**Concentration curves for 10 African cities, development over time.** Notes: * sample size <1000; ** sample size <500. A concentration curve above the line of equality indicates that child mortality is higher amongst the poor than amongst the rich. The level of inequality is higher if the distance between the concentration curve and the line of equality is greater.

Figure 2 illustrates relative inequalities across the ten included cities by means of concentration curves. For almost all surveys and all cities (with the exception of one survey each in Dar es Salaam and Accra), concentration curves always lie almost entirely above the lines of equality, indicating that mortality is higher in lower wealth quintiles than in higher wealth quintiles. In Dar es Salaam and Accra, concentration curves have an odd shape, which is likely to be related to the very low sample sizes in these cities.

For eight out of the ten included cities, two surveys were available to assess trends in the development of inequalities over time. In most cities, that is, Cairo, Lagos, Dar es Salaam, Nairobi and Dakar, inequalities appear to have decreased over time as concentration curves from more recent surveys lie closer to the lines of equality. In Abidjan, inequality appears to have increased as the concentration curve for 2011–2012 is farther away from the line of equality than the concentration curve for 1998–1999. In Addis Ababa and Accra, the concentration curves from earlier and later years cross, making it more difficult to determine whether inequalities increased or decreased over time.

### Comparison of inequality across cities and over time

Table 2 compares inequalities in child mortality rates across cities and over time using four measures of inequality: the difference between poor and rich, the ratio, the concentration index and the Erreygers index. In the earlier round of surveys, all four measures exhibit significant inequalities across all cities except Accra as 95% confidence intervals do not include zero (or one in the case of the ratio). However, when comparing inequalities across cities, confidence intervals (CI) of all measures almost always overlap. Notable exceptions include Cairo, where the difference in child mortality rates between rich and poor was 40 deaths (CI 6 to 75) per 1,000 childbirths in 2000, when compared with Dar es Salaam, where the difference was 196 deaths (CI 83 to 310) per 1,000 childbirths in 1999, and Abidjan, where both the concentration index and the Erreygers index are significantly smaller than these measures in Dar es Salaam. In addition, absolute inequality as measured by the Erreygers index is significantly lower in Cairo than in Dar es Salaam and Addis Ababa because the absolute difference between rich and poor in Cairo is relatively small. By contrast, the level of relative inequality as measured by the concentration index is not significantly different in Cairo because in relative terms richer children still die much more frequently than poor children.

**Table 2:** Comparison of the level of inequalities in child mortality and trends over time across ten African cities

		**Earlier surveys**			**More recent surveys**		
**City (years)**	**Measure of inequality**	**Value**	**95% confidence interval**		**Value**	**95% confidence interval**	
Cairo (2000, 2008)	Difference (poor minus rich)	40.48	5.72	75.25	14.12	−22.04	50.28
	Ratio (poor/rich)	2.98	1.10	8.10	1.44	0.56	3.72
	Concentration index	−0.18	−0.34	−0.02	−0.10	−0.27	0.06
	Erreyger’s index	−0.03	−0.05	0.00	−0.02	−0.04	0.01
Lagos (2003, 2008)	Difference (poor minus rich)	155.90	55.01	256.79	22.94	−31.51	77.40
	Ratio (poor/rich)	n/a	n/a	n/a	1.38	0.63	3.03
	Concentration index	−0.28	−0.45	−0.11	−0.04	−0.15	0.07
	Erreyger’s index	−0.11	−0.18	−0.04	−0.01	−0.05	0.03
Kinshasa (2007)	Difference (poor minus rich)	-	-	-	56.79	10.35	103.24
	Ratio (poor/rich)	-	-	-	1.83	1.11	3.04
	Concentration index	-	-	-	−0.11	−0.20	−0.03
	Erreyger’s index	-	-	-	−0.04	−0.08	−0.01
Luanda (2011)	Difference (poor minus rich)	-	-	-	42.20	9.86	74.53
	Ratio (poor/rich)	-	-	-	1.90	1.17	3.10
	Concentration index	-	-	-	−0.14	−0.22	−0.07
	Erreyger’s index	-	-	-	−0.04	−0.06	−0.02
Abidjan (1998–99, 2011–12)	Difference (poor minus rich)	62.61	2.14	123.08	107.80	54.99	160.61
	Ratio (poor/rich)	1.93	1.01	3.68	4.43	1.97	9.97
	Concentration index	−0.10	−0.19	0.00	−0.16	−0.26	−0.07
	Erreyger’s index	−0.05	−0.09	0.00	−0.06	−0.10	−0.02
Dar es Salaam (1999, 2010)	Difference (poor minus rich)	196.47	82.63	310.31	−20.05	−100.49	60.38
	Ratio (poor/rich)	12.30	1.64	92.23	0.69	0.16	3.03
	Concentration index	−0.34	−0.48	−0.20	−0.05	−0.21	0.11
	Erreyger’s index	−0.16	−0.23	−0.09	−0.02	−0.09	0.05
Nairobi (1998, 2008–09)	Difference (poor minus rich)	121.81	28.28	215.33	15.66	−31.49	62.82
	Ratio (poor/rich)	9.89	1.27	77.07	1.45	0.47	4.48
	Concentration index	−0.31	−0.52	−0.09	−0.04	−0.19	0.12
	Erreyger’s index	−0.09	−0.15	−0.03	−0.01	−0.04	0.03
Dakar (1997, 2010–11)	Difference (poor minus rich)	107.92	46.50	169.33	29.83	−9.64	69.30
	Ratio (poor/rich)	3.04	1.56	5.93	1.97	0.80	4.88
	Concentration index	−0.19	−0.31	−0.08	−0.11	−0.25	0.02
	Erreyger’s index	−0.07	−0.11	−0.03	−0.02	−0.05	0.00
Addis Ababa (2000, 2011)	Difference (poor minus rich)	96.39	31.58	161.20	57.97	5.46	110.49
	Ratio (poor/rich)	2.35	1.29	4.28	2.87	1.05	7.83
	Concentration index	−0.18	−0.27	−0.10	−0.21	−0.37	−0.04
	Erreyger’s index	−0.09	−0.14	−0.05	−0.04	−0.08	−0.01
Accra (1998, 2008)	Difference (poor minus rich)	8.53	−62.61	79.67	27.10	−29.41	83.61
	Ratio (poor/rich)	1.17	0.32	4.25	2.72	0.29	25.51
	Concentration index	−0.13	−0.40	0.13	−0.25	−0.46	−0.03
	Erreyger’s index	−0.03	−0.08	0.03	−0.05	−0.09	−0.01

Note: The difference and the Erreyger’s index are measures of absolute inequality, while the ratio and the concentration index measure relative inequality.

In the most recent surveys, significant inequalities exist in Kinshasa, Luanda, Abidjan, and Addis Ababa, where confidence intervals of all four inequality measures do not include zero (or one in the case of the ratio). In Accra, the concentration index and the Erreyger’s index also indicate significant inequalities. By contrast, when compared with the earlier surveys, measured inequality has reduced considerably in Cairo, Lagos, Dar es Salaam, Nairobi, and Dakar. In fact, inequality has become insignificant in these cities as confidence intervals now include zero (or one). For Dar es Salaam, the difference between the earlier and later surveys appears to be significant as confidence intervals of most measures do not overlap. By contrast, inequality seems to have increased in Abidjan and Accra although confidence intervals of earlier and later surveys overlap. In Addis Ababa, absolute inequality appears to have reduced while relative inequality remained more or less unchanged.

## Discussion

This is the first study to systematically investigate socio-economic inequalities in child mortality within and across African cities and their development over time. We disaggregated data from two rounds of demographic and health surveys and calculated four measures of socio-economic inequality for ten cities in Africa. The results show that in most cities, child mortality is considerably higher among the poor than among the rich, with the difference between the poorest quintile and the richest quintile reaching as much as 108 deaths per 1,000 live births in Abidjan in 2011–2012. Around the year 2000, Dar es Salaam had the highest level of inequality, while Abidjan and Cairo had rather low absolute (Cairo) and relative (Abidjan) inequality. Since then, inequality appears to have reduced in about half of the included cities (Cairo, Lagos, Dar es Salaam, Nairobi and Dakar), while it appears to have increased in Abidjan. However, given the high degree of uncertainty surrounding the point estimates of child mortality (see Figure 1) and the resulting uncertainty around our measures of inequality (Table 2), results need to be interpreted with caution.

The study has a number of limitations. The most important one is the limited sample size of DHS within cities, which are not intended to be used for within-city analyses. In the earlier round of surveys, only four cities had data available from more than 1,000 children, and also in the later round, the samples of three cities were smaller than 1,000 children (see Table 1). Consequently, reliability of child mortality estimates is questionable and large confidence intervals around point estimates complicate the interpretation of results (see Table 2). Nevertheless, DHS remain the most reliable source of information currently available in most African countries, and they are used regularly by international agencies for estimating child mortality [40]. Civil registration and vital statistics systems (CRVS) – if available at all – usually record only a small fraction of all births and deaths [41], and child mortality data from censuses is often questionable. For example, in Abidjan, it is estimated that only about 70% of births and less than 40% of deaths (and even fewer among children) are registered by the CRVS [42], and child mortality was grossly underreported in the last Ivorian census conducted in 1998 [43]. DHS usually report child mortality figures for children born in the five years preceding the survey [29]. Our study included birth histories and deaths of children born in the ten years preceding the survey in order to increase the sample size. This means that recent changes in inequality are only marginally reflected in our estimates, and they are not directly comparable with the figures reported in DHS. Furthermore, because birth histories cover the previous ten years, some of the included births and deaths may, in fact, have occurred before households moved to cities.

A second limitation of our study related to the use of DHS data is that the DHS wealth index combines information about household ownership of selected assets with the availability of basic community-level services, such as water or electricity [31]. This can be problematic because it can lead to the misclassification of relatively rich households into the group of relatively poor households if they live in relatively poor neighborhoods [44,45]. The problem is thought to be particularly relevant when comparing urban areas, where more community-level services are available, with rural areas. However, in our study of major cities, where residential patterns tend to be more segregated, the misclassification of households is likely to be less of a problem, although it may still lead to an underestimation of the true extent of inequality.

A third limitation of this study concerns the comparison of inequality across cities and the assessment of trends over time. One problem is that DHS data from different cities were not available for the same years. For example, the most recent survey from Cairo was from 2008, while the most recent survey from Luanda was from 2011. Consequently, inequality is compared across cities at different points in time, and depending on the trend over time differences in inequality across cities might have reduced or increased beyond what can be seen in the data. In addition, the time period between the first and the second survey ranged from five years in Lagos to almost fourteen years in Abidjan and Dakar, and consequently, the change in inequality from the first survey to the later survey, which is shown in Figure 2 and Table 2, might appear relatively smaller in cities with a shorter time period between surveys than in those with longer periods. Another problem is that we selected only four measures of inequality to compare cities and to assess the trend in inequalities over time. A host of further measures are available [27,28,33], including odds ratios, the slope index of inequality, the relative index of inequality, the generalized concentration index, and the Wagstaff index. In addition, it has been shown that measured inequality may differ depending on the chosen indicator [12,14,36]. However, our selection of two measures of relative inequality (the rate ratio and the concentration index) and two measures of absolute inequality (the difference and the Erreygers index) is similar to other studies [5] and should provide a reasonably nuanced view of the development of inequalities across cities.

Finally, a major weakness of our study is that it did not investigate the underlying reasons for the identified differences in inequalities across cities and for the differences in trends over time. Prior studies have decomposed calculated concentration indices in order to assess the contribution of different factors to inequality in infant [46] or child [47-49] mortality. However, the small size of the samples from the 10 African cities included in our study would draw into question the value of such analyses. Further research is needed to improve data availability from cities and to investigate reasons for differences in inequality and differences in trends over time.

Despite these limitations, our research has important implications for policy-makers and researchers. In 2010, a joint WHO and UN-HABITAT report [21] urged countries to unmask health-related inequities in cities. Our study is the first to do this and to show the high level of inequalities in child mortality that exists within African cities. However, both average child mortality in the included cities and mortality of the poorest quintile are generally considerably below the corresponding national figures (see Additional file 1: Table S1 in the supplementary online material for national child mortality rates by wealth quintile). A previous study [20] found that in nine low income countries, child mortality was significantly higher among the urban poor than among the rural poor. Our sample included no cities from these nine countries but our results seem to suggest that child mortality is lower in major African cities than in the rest of the country (see Additional file 1: Table S1), not only on average but also among the poor. This is in line with findings of another recent study [9], which found that child mortality rates of children living in households in urban slums are higher than the rates of those living in formal settlements – but still lower than child mortality rates in rural areas.

In addition, our study reveals that the level of inequality in child mortality differs across cities and over time. Inequality in Abidjan was relatively low in 1998–1999, when compared with other African cities, but it was rather high in 2011–2012. Inequality in Cairo, Lagos, Dar es Salaam, Nairobi and Dakar seems to have reduced over (more or less) the same period of time. One explanation for these opposing trends might be that Abidjan suffered considerably during the time of political instability and civil war in Côte d’Ivoire, which lasted from 1999 to 2011 [50]. Armed conflict has been shown to contribute significantly to increased child mortality not only during but also after the period of active warfare [51-53]. Conflict is likely to disproportionately affect the poor [54], and consequently inequities seem to be particularly severe in countries with a history of recent conflict [55]. Another possible explanation might be that inequality of wealth decreased in some cities, and that this drove a decrease in inequality of child mortality. A decrease in inequality of mean wealth scores by income quintiles in Dar es Salaam, Lagos and Nairobi – cities where inequality seems to have decreased over time – might support this hypothesis, although there is no clear correlation, when looking at all included cities (see Additional file 1: Table S2). Child mortality is, of course, affected also by more proximate factors, including lack of shelter, sufficient nutrition or access to clean water and sanitation, as well as inadequate public health expenditures, low female education and short birth intervals [56-58], and inequality in child mortality is largely related to unequal distribution of these factors [46,48,49]. Also, Kinshasa, Luanda, Addis Ababa and Accra were found to have significant levels of inequality in the more recent round of surveys, and these cities may benefit from looking at cities that achieved reductions in inequality over time.

Finally, our study shows that larger sample sizes are needed in order to more reliably assess inequalities in child mortality within and across cities and in their development over time. Luanda was the only city included in this study with a DHS that contained full birth histories for more than 2,000 children (Table 1). WHO and UNHABITAT have asked countries to disaggregate available data in order to reveal health-related inequalities within cities [21]. However, the small sample sizes of DHS mean that there is considerable uncertainty when this data is disaggregated in order to assess inequalities within cities. The need to improve data availability in developing countries has been recognized by the most important players in global health [59]. Ultimately, civil registration and vital statistics systems will need to be strengthened. However, in the short-term coverage of health and demographic surveillance systems, which collect demographic and health data for a population living in a well-defined geographic area, could be improved in cities, whereas they are currently mostly focused on rural areas [60,61].

## Conclusions

The need to investigate health-related inequalities within cities is increasingly being recognized by international agencies [16,21] as this is a prerequisite for national governments to act against them. Our study shows that considerable inequalities in child mortality exist in almost all cities but that the level of inequalities and their development over time differ across cities. This implies that inequalities are amenable to policy interventions and that it is worth investigating why inequalities are higher in one city than in another. However, the size of the data samples available from our cities is relatively small, leading to considerable uncertainty concerning the rate of child mortality in different population groups (poor versus rich) and this complicates interpretation of our results. Larger sample sizes are needed in order to improve the certainty of our results, to reliably quantify the level of inequalities within cities and to identify factors that can help to reduce inequalities over time.

## Competing interests

The authors declare that they have no competing interests.

## Authors’ contributions

WQ and LS conceptualized the study, which was then designed in collaboration by all authors. WQ, JA, KD and MTB assured data collection in Côte d’Ivoire. All authors helped to interpret the data from their respective cities. WQ drafted the manuscript. All authors critically revised and approved the final manuscript.

## Pre-publication history

The pre-publication history for this paper can be accessed here:

http://www.biomedcentral.com/1741-7015/12/95/prepub

## Supplementary Material

Additional file 1 **Box S1.** On the DHS sampling procedure and the use of sample weights. **Table S1.** National child mortality rates in countries and DHS surveys included in this study. **Table S2.** Mean wealth index scores by income quintile across 10 African cities. Click here for file
