# Supplementary material for: Inequalities in child mortality in ten major African cities
Source: BMC Med. 2014 Jun 6;12:95. doi: 10.1186/1741-7015-12-95 (PMC4066831; doi:10.1186/1741-7015-12-95)
Supplement: Additional file 1 — Box S1. On the DHS sampling procedure and the use of sample weights. Table S1. National child mortality rates in countries and DHS surveys included in this study. Table S2. Mean wealth index scores by income quintile across 10 African cities. [file 1741-7015-12-95-S1.docx]

**Supplementary online material**

**Box S1: On the DHS sampling procedure and the use of sample weights**

DHS surveys are, in general, based on a two-stage cluster sampling procedure [30]. In the first stage, a stratified sample of Enumeration Areas (EAs) is selected and in the second stage, a sample of households is selected within each EA. This cluster sampling procedure often means that individual households face differential probabilities of being selected, and sample weights have to be used in order to adjust for this (and for differences in response rates) and to assure representativeness for the whole country.

However, a particularity of DHS surveys is that samples are usually self-weighting at a defined sample domain (e.g. a region). This is achieved by adjusting the number of households selected per EA (i.e. a larger number of households is selected from larger EAs and a smaller number from smaller EAs). Consequently, every household within the sample domain (e.g. the region) has the same probability of being selected and sample weights do not need to be used.

The surveys included in our study often define one sample domain that is identical to the city of interest, or the city is located wholly within one sample domain. This means that each sampled household within the included cities has the same probability of being selected for the sample.

According to the guide to DHS statistics [29], the relatively small differences in response rates usually do not need to be adjusted for. In addition, the use of sample weights could bias estimates of confidence intervals because most statistical software packages (including SPSS) use the number of weighted cases to produce the confidence intervals instead of the true number of observations. Therefore, we decided not to use sample weights for our analyses.

**Table S1: National child mortality rates in countries and DHS surveys included in this study**

|  | **Q1** | **Q2** | **Q3** | **Q4** | **Q5** | **Average** |
| --- | --- | --- | --- | --- | --- | --- |
| **Angola** |  |  |  |  |  |  |
| 2011 | 107 | 111 | 120 | 102 | 83 | 102 |
| **Egypt** |  |  |  |  |  |  |
| 2000 | 98 | 80 | 69 | 52 | 33 | 66 |
| 2008 | 48 | 36 | 32 | 26 | 18 | 32 |
| **Côte d'Ivoire** | |  |  |  |  |  |
| 1998 | 231 | 188 | 168 | 157 | 83 | 166 |
| 2011-12 | 123 | 129 | 124 | 108 | 82 | 116 |
| **Democratic Republic of the Congo** | | |  |  |  |  |
| 2007 | 181 | 176 | 154 | 151 | 95 | 151 |
| **Ethiopia** |  |  |  |  |  |  |
| 2000 | 159 | 194 | 226 | 206 | 147 | 186 |
| 2011 | 136 | 120 | 97 | 98 | 83 | 107 |
| **Ghana** |  |  |  |  |  |  |
| 1998 | 134 | 120 | 119 | 98 | 46 | 104 |
| 2008 | 102 | 77 | 102 | 67 | 59 | 81 |
| **Kenya** |  |  |  |  |  |  |
| 1998 | 137 | 131 | 92 | 83 | 61 | 101 |
| 2008 | 96 | 102 | 91 | 50 | 69 | 82 |
| **Nigeria** |  |  |  |  |  |  |
| 2003 | 253 | 294 | 215 | 177 | 79 | 204 |
| 2008 | 217 | 211 | 164 | 129 | 87 | 162 |
| **Senegal** |  |  |  |  |  |  |
| 1997 | 180 | 179 | 144 | 102 | 70 | 135 |
| 2010 | 117 | 92 | 79 | 68 | 54 | 82 |
| **United Republic of Tanzania** | | |  |  |  |  |
| 1999 | 159 | 161 | 193 | 155 | 134 | 161 |
| 2010 | 103 | 92 | 90 | 86 | 84 | 91 |

Notes: Q1 = quintile 1 = poorest; Q5 = quintile 5 = richest.

Source: National DHS surveys and WHO (2014) [61]

**Table S2: Mean wealth index scores by income quintile across ten African cities.**

|  |  | **Quintile** | | | | |
| --- | --- | --- | --- | --- | --- | --- |
|  | **Year** | **1** | **2** | **3** | **4** | **5** |
| **Cairo** | *2000* | -0.22 | 0.52 | 0.91 | 1.19 | 1.50 |
|  | *2008* | -0.33 | 0.32 | 0.71 | 1.14 | 1.86 |
| **Lagos** | *2003* | 0.88 | 1.37 | 1.67 | 1.96 | 2.49 |
|  | *2008* | 0.94 | 1.33 | 1.52 | 1.75 | 2.24 |
| **Kinshasa** | *2007* | 0.43 | 1.37 | 1.86 | 2.25 | 2.76 |
| **Luanda** | *2011* | 0.71 | 1.02 | 1.14 | 1.27 | 1.51 |
| **Abidjan** | *1998-99* | -0.35 | 0.16 | 0.48 | 0.97 | 1.94 |
|  | *2011-12* | 0.20 | 0.58 | 0.87 | 1.28 | 2.14 |
| **Dar es Salaam** | *1999* | 0.31 | 0.72 | 1.02 | 1.58 | 2.87 |
|  | *2010* | 0.6 | 1 | 1.57 | 2.07 | 2.85 |
| **Nairobi** | *1998* | 0.01 | 0.74 | 1.29 | 1.98 | 3.81 |
|  | *2008-09* | 0.75 | 1.33 | 1.56 | 1.82 | 2.26 |
| **Dakar** | *1997* | -0.02 | 0.54 | 1.06 | 1.93 | 3.37 |
|  | *2010-11* | 0.68 | 1.14 | 1.37 | 1.65 | 2.09 |
| **Addis Ababa** | *2000* | 0.95 | 1.61 | 1.96 | 2.30 | 2.79 |
|  | *2011* | 0.68 | 1.36 | 1.73 | 2.08 | 2.54 |
| **Accra** | *1998* | 0.2 | 0.89 | 1.49 | 2.02 | 2.59 |
|  | *2008* | 0.19 | 0.82 | 1.18 | 1.52 | 2.28 |

Note: National means of the wealth index scores are always close to zero as they are standardized during calculations to produce z-scores. Consequently, the wealth index scores themselves are not comparable over time and across cities. This means that the data in the table below do not suggest, for example, that the richest quintile in Nairobi became poorer in absolute terms between 1998 and 2008-09. However, the data can be interpreted in a relative way, i.e. the richest quintile in Nairobi in 2008-09 was less rich in relative terms, which may indicate that the situation of the poor improved.
